# Butyrate and Butyrate-Producing Bacteria in Cardiovascular–Kidney–Metabolic Syndrome

**DOI:** 10.3390/antiox15070812

**Published:** 2026-06-28

**Authors:** Wenli Huang, Fen Zhou, Shuo Wang, Meng Shu, Zhongchun Liu, Ling Gao

**Affiliations:** 1 Department of Endocrinology and Metabolism, Renmin Hospital of Wuhan University, Wuhan 430060, China; wenlihuang@link.cuhk.edu.hk (W.H.); rm002668@whu.edu.cn (F.Z.); shuowang.rm@whu.edu.cn (S.W.); rm004373@whu.edu.cn (M.S.); 2 Department of Psychiatry, Renmin Hospital of Wuhan University, Wuhan 430060, China; 3 Taikang Center for Life and Medical Sciences, Wuhan University, Wuhan 430060, China; 4 State Key Laboratory of Metabolism and Regulation in Complex Organisms, Wuhan University, Wuhan 430060, China

**Keywords:** butyrate, butyrate-producing bacteria, CKM syndrome, gut microbiota dysbiosis

## Abstract

The recently conceptualized Cardiovascular–Kidney–Metabolic (CKM) syndrome represents a pressing global health burden, characterized by a vicious cycle of dysfunction among the cardiac, renal, and metabolic systems. Growing evidence suggests that gut microbiota dysbiosis, specifically, a loss of butyrate-producing bacteria (BPB) and the resulting systemic butyrate deficiency, may be an important but previously overlooked driver of CKM progression. In this review, we synthesize available evidence linking butyrate to the integrated, multi-organ pathophysiology of CKM and propose a conceptual framework we term the gut-butyrate-CKM axis. We discuss the multiple mechanisms by which butyrate and BPB exert protective effects, including targeting key pathophysiological features of CKM, such as insulin resistance (IR), metabolic inflammation, oxidative stress, endothelial dysfunction, renin–angiotensin–aldosterone system (RAAS) overactivation, and gut dysbiosis itself. Through a critical appraisal of human studies, we bring together findings from direct butyrate supplementation, dietary interventions, and microbiota-directed strategies. Based on this, we argue that butyrate serves as a central hub linking gut homeostasis to systemic metabolic and cardiorenal health. By integrating previously fragmented observations into a coherent framework, this review addresses a conceptual gap in our understanding of CKM pathogenesis and points to actionable, microbiota-targeted therapeutic strategies that could help break the disease cycle. Given the current lack of integrated management options for CKM, our work offers insights for future translational research and clinical practice, highlighting butyrate-centered approaches as a potential paradigm shift in CKM care.

## 1. Introduction

The escalating global burden of non-communicable diseases has converged into a significant public health crisis, epitomized by the recent conceptualization of CKM syndrome [1]. This syndrome delineates a pathophysiological interplay between obesity, type 2 diabetes (T2D), chronic kidney disease (CKD), and cardiovascular disease (CVD), creating a self-perpetuating cycle of multi-organ dysfunction. The prevalence of CKM syndrome is reaching pandemic proportions, imposing an unprecedented burden on healthcare systems worldwide. Its pathogenesis is multifaceted, driven by a constellation of interrelated processes including chronic low-grade inflammation, oxidative stress, endothelial dysfunction, overactivation of the RAAS, and IR [2].

While these classical pathways are well-established, a paradigm shift has emerged with the recognition of the gut microbiota as a critical, system-wide modulator. Gut dysbiosis is now implicated not merely as a bystander but as an active disruptor of systemic homeostasis, influencing each core pathological component of CKM [3,4]. Among the array of microbial metabolites, short-chain fatty acids (SCFAs), particularly butyrate, have garnered significant attention. This four-carbon acid, primarily produced by the fermentation of dietary fiber by colonic bacteria, transcends its role as a local energy source for colonocytes [5]. Its mechanisms of action are remarkably diverse, encompassing epigenetic regulation via inhibition of histone deacetylases (HDACs), modulation of cellular signaling via G protein-coupled receptor (GPCR) activation (e.g., GPR41, GPR43, GPR109a), and reshaping the gut microbial ecosystem towards a more eubiotic state [6,7]. Consequently, the decline in BPB (e.g., *Faecalibacterium prausnitzii*, *Roseburia* spp.) and the subsequent reduction in butyrate availability represent a compelling etiological axis in CKM syndrome [8,9,10,11,12].

Despite this burgeoning evidence, a critical gap persists. Existing reviews often adopt a siloed perspective, focusing on individual diseases within the CKM spectrum, such as isolated CVD or diabetes, or on the general role of SCFAs. They fail to capture the holistic, cross-organ perspective demanded by the syndrome’s interconnected nature and lack a dedicated synthesis directly linking butyrate and BPB to the integrated pathophysiology of CKM. This gap underscores the necessity for a comprehensive review that moves beyond a catalog of effects in single organs to propose a unified framework.

Therefore, this review aims to bridge this conceptual and mechanistic divide. We first elucidate the current epidemiological landscape and the complex, networked pathophysiology of CKM syndrome, highlighting the central role of its core mediators. We then present an integrative analysis of the multifaceted molecular mechanisms of butyrate, demonstrating how its diverse actions converge to disrupt key pathogenic cycles. Subsequently, we critically appraise the clinical and preclinical evidence for butyrate’s protective effects across cardiovascular, renal, and metabolic compartments, evaluating potential therapeutic strategies from direct supplementation to dietary and microbial interventions. By synthesizing these fragmented insights into a cohesive gut-butyrate-CKM axis framework, this review seeks to establish butyrate as a central molecular and ecological link between intestinal health and systemic multi-organ dysfunction. Distinct from earlier gut-cardiorenal-metabolic interaction models, this unified gut-butyrate-CKM axis resolves their prominent limitations. Classic reviews tend to regard toxic microbial metabolites as core pathogenic mediators, and existing butyrate investigations are confined to individual cardiac, renal or metabolic phenotypes, lacking systematic integration under the CKM syndrome framework. In doing so, we aim to not only advance the mechanistic understanding of CKM pathogenesis but also to illuminate novel, microbiota-targeted avenues with tangible potential for prevention and therapeutic intervention.

## 2. Current Understanding of CKM Syndrome

### 2.1. Epidemiological Burden

CKM syndrome represents a major and growing global public health burden. Epidemiological studies indicate that CKD affects 10–20% of the global population, with approximately 5 million individuals requiring renal replacement therapy [13]. In China, CKM syndrome affects 42.6–71.0% of middle-aged and older adults [14]. In the United States, nearly 90% of adults exhibit at least one CKM risk factor, such as hypertension, dyslipidemia, or impaired renal function, and only about 10% maintain ideal cardiorenal metabolic health [15].

The economic toll is equally striking. In China, CKM-related medical expenditures account for 28% of total healthcare spending, with direct costs for cardiovascular and cerebrovascular diseases exceeding RMB 540.6 billion annually as of 2017 [16]. In the United States, the cost of CVD was an estimated $407.3 billion in 2018 to 2019, and dialysis for end-stage renal disease (ESRD) costs about $25.3 billion in 2019 [17].

The American Heart Association’s 2023 staging framework stratifies CKM from stage 0 (no risk factors) through stage 4 (clinical CVD with persistent metabolic dysfunction), informing stage-specific interventions [18]. This staging system helps stratify prognosis: all-cause mortality rises sharply with advancing stage, increasing by 36%, 150%, and 300% in Stages 2, 3, and 4, respectively, compared to Stage 0. Coexisting cardiovascular, metabolic, and kidney disorders synergistically accelerate end-organ damage and mortality risk [19]. With aging populations and rising obesity prevalence, CKM syndrome prevalence is expected to grow, posing ongoing challenges to health systems worldwide.

### 2.2. Risk Factors

The onset and progression of CKM syndrome result from a complex interplay between traditional metabolic risk factors and emerging risk determinants.

Traditional risk factors form the core of CKM syndrome pathophysiology, including overweight/obesity (particularly central obesity), IR, hypertension, dyslipidemia (e.g., hypertriglyceridemia, low high-density lipoprotein cholesterol (HDL-C) level), and hyperglycemia [20]. These factors do not act in isolation but interact through shared mechanisms, such as oxidative stress, chronic inflammation, and endothelial dysfunction, creating a vicious cycle that accelerates multi-organs damage [2]. Obesity plays a central driving role: excess visceral adipose tissue secretes pro-inflammatory cytokines, induces lipotoxicity, and releases angiotensinogen, thereby impairing cardiac and renal function while exacerbating IR.

Risk-enhancing factors may independently or synergistically accelerate CKM progression [18]. These include chronic inflammatory conditions (e.g., psoriasis, rheumatoid arthritis, systemic lupus erythematosus, and HIV/AIDS), sleep disorders which exacerbate metabolic and cardiovascular dysfunction via intermittent hypoxia and sleep fragmentation, sex-specific risks (early menopause, adverse pregnancy outcomes, polycystic ovary syndrome in women, and erectile dysfunction in men), and specific biomarkers, such as high-sensitivity C-reactive protein (hs-CRP ≥ 2.0 mg/L), a key indicator of systemic inflammation that has been validated as a robust predictor of CKM syndrome development and progression [18].

Socio-ecological factors constitute the underlying network contributing to CKM syndrome pathogenesis. low socioeconomic status, limited education, unhealthy dietary patterns, sedentary lifestyle, smoking, and poor sleep quality collectively shape the trajectory from health to disease [18,21,22].

### 2.3. Pathophysiology: An Integrated Network of Interconnected Mechanisms

The essence of CKM syndrome lies in a self-perpetuating cycle among metabolic dysregulation, kidney injury, and CVD. Six core mechanisms, including IR, chronic inflammation, oxidative stress, endothelial dysfunction, overactivation of the RAAS, and gut microbiota dysbiosis, interact through multiple feedback loops [2], ultimately leading to end-organ damage. Notably, these mechanisms are not independent. They converge and amplify one another, creating a pathogenic network that is more than the sum of its parts.

#### 2.3.1. Insulin Resistance

IR serves as the central pathophysiological hub of CKM syndrome [23,24]. Through complex direct and indirect mechanisms, it causes damage to the heart, kidneys, and metabolic system, thereby regulating the initiation and progression of CKM syndrome [25].

The pathogenesis originates in the excessive accumulation of visceral adipose tissue, which secretes abundant pro-inflammatory cytokines, including tumor necrosis factor-alpha (TNF-α) and interleukin-6 (IL-6) [26]. This process instigates a state of systemic chronic low-grade inflammation and enhances lipolysis, leading to elevated circulating free fatty acids [27]. The subsequent ectopic deposition of free fatty acids in non-adipose organs, such as the liver and skeletal muscle, exerts potent lipotoxic effects that further impair insulin signaling, thereby establishing a self-perpetuating vicious cycle. Concomitantly, impaired glucose uptake and utilization in insulin-responsive tissues, when coupled with enhanced hepatic gluconeogenesis, perpetuates a hyperglycemic state. This metabolic milieu, characterized by chronic inflammation and hyperinsulinemia, promotes activation of the sympathetic nervous system and the RAAS, resulting in vasoconstriction, hypertension, and sodium retention [28]. These changes directly accelerate end-organ damage, including myocardial hypertrophy and renal fibrosis.

In the cardiovascular system, IR induces endothelial dysfunction, accelerates atherogenesis, and drives lipotoxicity-mediated metabolic remodeling in cardiomyocytes [25,29]. Indirectly, IR fosters hypertension, atherogenic dyslipidemia, and persistent systemic inflammation, amplifying the risk of major adverse cardiovascular events such as coronary artery disease and heart failure [29,30]. In the kidney, IR propagates injury via intertwined hemodynamic and metabolic routes [31,32]. Hemodynamically, impaired insulin-mediated vasodilation predisposes to aberrant efferent arteriolar constriction, leading to glomerular hypertension, hyperfiltration, and hyperperfusion, a pivotal early mechanism in glomerular injury [31,33]. Metabolically, hyperinsulinemia stimulates renal sodium reabsorption, exacerbating volume overload and hypertension, while also promoting dyslipidemia and enhanced uric acid reabsorption, thereby creating a profibrotic and pro-sclerotic renal environment [34,35,36]. Critically, a bidirectional, self-reinforcing cycle emerges between CKD and IR: declining renal function leads to the accumulation of uremic toxins, chronic inflammation, and metabolic acidosis, which further impair insulin sensitivity, whereas diminished insulin clearance exacerbates hyperinsulinemia, perpetuating a feed-forward loop that accelerates disease progression [37].

#### 2.3.2. Metabolic Inflammation

Metabolic inflammation is a low-grade chronic inflammation initiated by dysfunctional visceral adipose tissue. Under metabolic stress (e.g., obesity), adipose tissue transforms into an inflammatory secretory organ, releasing TNF-α and IL-6 that activate nuclear factor kappa-B (NF-κB), NOD-like receptor thermal protein domain associated protein 3 (NLRP3) inflammasome, JAK-STAT, PI3K-AKT pathways, forming a complex inflammatory network that exacerbates IR and contributes to endothelial dysfunction, myocardial fibrosis, and glomerulosclerosis [38,39,40,41].

Metabolically, inflammatory factors induce IR by interfering with insulin signaling, and IR further stimulates adipose inflammation, creating an initial disease-promoting cycle [38,42]. Cardiovascularly, it accelerates atherosclerosis (via monocyte recruitment and foam cell formation) and exerts direct lipotoxicity on cardiomyocytes (via epicardial adipose-derived fatty acids and inflammatory factors), causing myocardial metabolic disorders and fibrosis [43,44,45]. Renally, inflammatory factor-mediated immune cell infiltration activates mesangial and renal tubular epithelial cells, initiating profibrotic pathways, promoting extracellular matrix deposition and basement membrane damage, and driving progressive renal function loss [46,47,48,49].

#### 2.3.3. Oxidative Stress

Oxidative stress interacts synergistically with IR and metabolic inflammation [50]. In metabolic disorders, mitochondrial dysfunction in adipose tissue, liver, and vascular endothelium generates excessive reactive oxygen species (ROS) [51,52]. Persistent hyperglycemia also promotes the formation of advanced glycation end products, which activate downstream inflammatory signaling via the receptor for advanced glycation end products, establishing a vicious cycle in which oxidative stress and chronic inflammation mutually amplify each other [53,54]. Excessive ROS induces lipid peroxidation, protein dysfunction, and DNA damage, directly compromising cellular structural integrity and activating inflammatory cascades [55]. These changes contribute to multidimensional impairments in insulin signal transduction, vascular endothelial function, myocardial metabolic homeostasis, and the renal filtration barrier [20,56,57,58].

#### 2.3.4. Endothelial Dysfunction

Endothelial dysfunction bridges metabolic disturbances with cardiovascular and renal injury. As the innermost vascular layer, the endothelium regulates vascular tone, permeability, platelet aggregation, and inflammation [59]. Endothelial dysfunction involves a complex network of interacting pathways, with oxidative stress and inflammatory signaling playing central roles. Obesity triggers redox imbalance in renal preglomerular arteries through the mitochondrial ROS-NADPH oxidase 4 signaling axis, leading to endothelial disruption and endoplasmic reticulum stress, ultimately promoting structural kidney damage [60]. Simultaneously, uremic toxins such as indoxyl sulfate activate the NEK7/NLRP3 inflammasome pathway, driving IL-1β release and inflammatory injury in the vascular endothelium [61]. Notably, endothelial cells exhibit spatiotemporal proliferative heterogeneity across organs (e.g., higher proliferative activity in specific cardiac regions), which may explain differential organ damage severity in CKM syndrome [5].

#### 2.3.5. RAAS Activation

RAAS is a key regulator of fluid balance and blood pressure. Its overactivation mediates cardiorenal damage through multiple pathways while engaging in close crosstalk with IR, oxidative stress, and endothelial dysfunction. RAAS overactivation and IR form a bidirectional cycle: Ang II impairs insulin signaling by inhibiting insulin receptor substrate phosphorylation, while secondary hyperinsulinemia further stimulates RAAS activity [62]. In the cardiovascular system, RAAS activation not only promotes hypertension and atherosclerosis through dysregulation of vascular tone and oxidative stress but also directly induces cardiomyocyte hypertrophy, collagen deposition, and electrophysiological remodeling, emerging as a central driver of heart failure progression [63,64,65]. In the renal domain, RAAS overactivation causes hemodynamic disturbances characterized by high pressure, high filtration, and high perfusion via imbalanced regulation of afferent and efferent arteriolar tone, and upregulates transforming growth factor-beta signaling, leading to excessive extracellular matrix deposition [66,67]. Notably, a newly identified mechanism in which cardiac-derived inflammatory factors activate the renal toll-like receptor 4 (TLR4)/NF-κB pathway under RAAS involvement provides a molecular explanation for cardiorenal cross-talk injury [68].

#### 2.3.6. Gut Microbiota Dysbiosis

Gut microbiota dysbiosis contributes to CKM syndrome pathogenesis and progression through three interrelated core pathways: metabolic imbalance, intestinal barrier disruption, and circadian rhythm disturbance [3]. Reduced abundance of BPB (e.g., *Ruminococcus*) leads to decreased renal histone H3 lysine 9 butyrylation, downregulating the expression of the protective gene MAS1 and compromising renal defense against hypertension [69]. Gut microbiota, such as *Escherichia coli*, convert tryptophan to indoxyl sulfate, which accumulates in CKD and disrupts cardiomyocyte mitochondrial function via the AHR/CYP1B1 pathway, thereby promoting heart failure [70,71]. Other gut microbiota-derived metabolites, including trimethylamine N-oxide (TMAO) and phenylacetylglutamine, elevate cardiovascular risk by promoting atherosclerotic plaque formation and enhancing platelet reactivity [72,73].

Dysbiosis also compromises the intestinal epithelial barrier, facilitating endotoxin (e.g., lipopolysaccharide, LPS) translocation and triggering chronic low-grade inflammation [74]. Translocated LPS forms complexes with LPS-binding protein and CD14 to activate the TLR4-myeloid differentiation factor-2 receptor complex on innate immune cells, which initiates MyD88/NF-κB inflammatory signaling and drives sustained release of pro-inflammatory cytokines, ultimately establishing chronic low-grade inflammation [75,76]. This persistent inflammatory milieu directly promotes IR, accelerates atherosclerosis through innate immune activation, and drives renal interstitial fibrosis [77]. Emerging evidence further highlights circadian misalignment as a novel pathway linking gut microbiota to CKM syndrome, where disruption of diurnal oscillations of microbial metabolites may destabilize atherosclerotic plaques and explain the characteristic morning peak in cardiovascular events [78,79].

**Figure 1:**
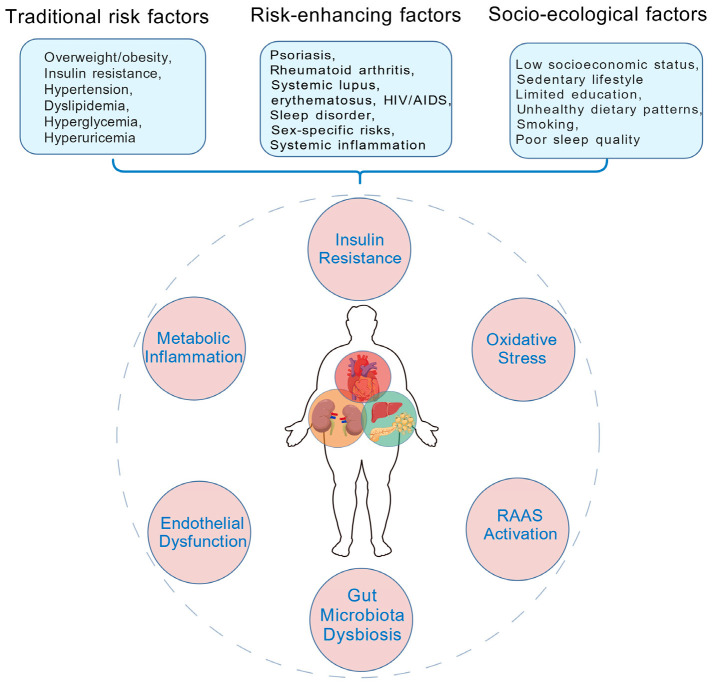
Risk factors and Pathophysiology of CKM syndrome.

In summary, the six core mechanisms, including IR, metabolic inflammation, oxidative stress, endothelial dysfunction, RAAS overactivation, and gut dysbiosis, do not operate in isolation. They form multiple vicious cycles (e.g., IR-inflammation-oxidative stress, gut dysbiosis-systemic inflammation) that synergistically promote cardiorenal and metabolic damage (Figure 1). This integrated network provides a rationale for multi-target therapeutic strategies, including those targeting butyrate and BPB, which will be discussed in subsequent sections.

## 3. Butyrate as a Multi-Target Therapeutic Agent in CKM Syndrome

**Figure 2:**
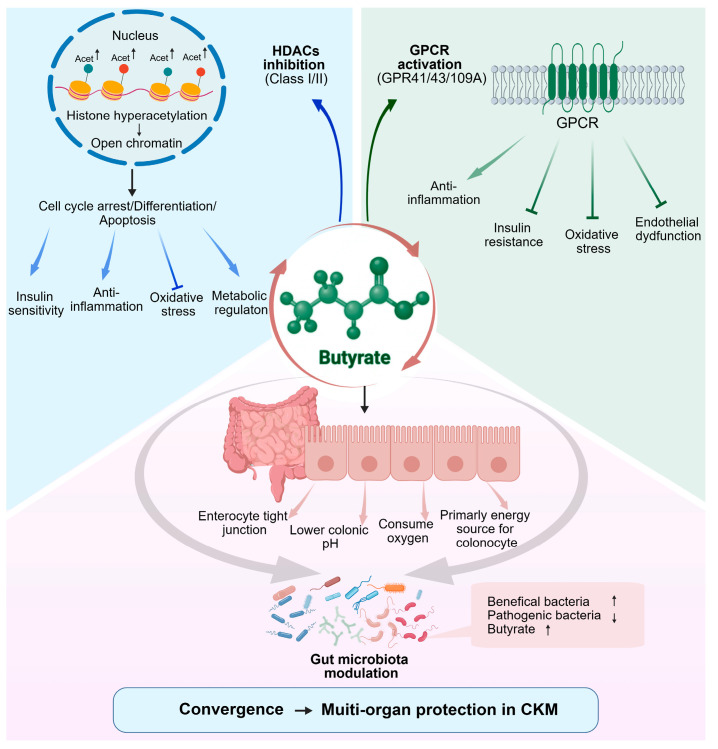
Butyrate Cellular Regulation Pathways. This synergistic multi-pathway regulatory network positions butyrate as a central regulatory hub for maintaining host health and a promising therapeutic target against CKM syndrome. ↓: Denotes a decreasing trend; ↑: Denotes an increasing trend.

Butyrate, a key microbial metabolite derived from dietary fiber fermentation, is a critical regulator at the intersection of the gut microbiome and host physiology. Its pleiotropic health benefits are orchestrated through three interconnected primary mechanisms: acting as an epigenetic modulator via HDACs inhibition, functioning as a signaling molecule through specific GPCRs, and fundamentally reshaping the gut microbial ecosystem. Critically, these pathways do not operate in isolation but converge to disrupt the core pathophysiological cycles of CKM (Figure 2).

### 3.1. Core Mechanisms of Butyrate Action

#### 3.1.1. Epigenetic Regulation via HDAC Inhibition

Butyrate is a potent inhibitor of Class I and II Zn^2+^-dependent HDACs, enzymes that remove acetyl groups from histones, leading to chromatin condensation and gene repression [80,81]. By inhibiting HDACs, butyrate promotes a hyperacetylated state of histones, facilitating a more open chromatin structure and the transcriptional activation of genes involved in cell cycle arrest, differentiation, and apoptosis [82,83]. The inhibition of specific HDACs underlies its organ-protective effects: HDAC1/2 suppression correlates with relieved inflammation and oxidative stress and renal protection [84,85]; HDAC3/4 inhibition is related to increased hepatokine fibroblast growth factor 21 to normalize systemic lipid metabolism and regulate the DNA damage response pathway to curb hepatocellular carcinoma cell proliferation [86,87]; HDAC5/6/8 inhibition is associated with attenuated cardiac hypertrophy and fibrotic responses [88,89,90]. Its nuanced interaction with Sirtuins (NAD^+^-dependent HDACs) highlights a context-dependent regulatory layer, underscoring the complexity and tissue specificity of butyrate’s epigenetic actions [91,92,93,94].

#### 3.1.2. Cell Signaling Through GPCR Activation

As an extracellular signaling molecule, butyrate activates specific GPCRs (GPR41, GPR43, GPR109A), mediating systemic anti-inflammatory and metabolic effects [95,96,97,98]. GPR41 activation is implicated in improving hepatic steatosis and ischemic stroke outcomes [97,99]. GPR43 activation is a cornerstone for immunomodulation and glucoregulation, enhancing glycogen synthesis and mitochondrial function [100,101]. GPR109A activation is vital for maintaining colonic anti-inflammatory tone and epithelial integrity [95,102,103]. The collective activation of these receptors on endothelial, immune, and metabolic cells directly counteracts IR, oxidative stress, and endothelial dysfunction [98,104,105].

#### 3.1.3. Reshaping the Gut Microbial Ecosystem

Butyrate fosters a eubiotic gut environment through multiple ecological strategies. Butyrate stimulates paneth cells to secrete antimicrobial peptides (e.g., α-defensins), selectively inhibiting pathogens, such as *Salmonella typhimurium*, Acinetobacter baumannii, and *Enterococcus faecium*, while promoting beneficial bacteria like *Akkermansia muciniphila*, *Bifidobacterium adolescentis*, and *Bifidobacterium longum* [6,106,107]. Additionally, butyrate improved leaky gut by upregulating the expression of the enterocyte tight junction protein and inhibiting inflammation [108,109,110,111]. As the name suggests, leaky gut refers to gut barrier dysfunction and impaired intestinal mucosal integrity induced by various diseases [112]. Of note, restored intestinal epithelial barrier by butyrate supplementation restrained bacterial translocation and improved gut bacterial composition [111]. In addition, as a weak acid and the primary energy source for colonocytes, butyrate lowers colonic pH and consumes oxygen, creating an anaerobic environment that favors the growth of strictly anaerobic BPB (e.g., *Roseburia intestinalis*, *Eubacterium rectale*, *F. prausnitzii*, and *Bacteroides* spp.) while inhibiting facultative anaerobes and pathobionts [113,114,115,116,117,118,119].

#### 3.1.4. Convergence of Butyrate’s Three Major Pathways in CKM Syndrome

Notably, these pathways mentioned above are not independent. They converge and reinforce each other, and this convergence is likely why butyrate can simultaneously affect multiple pathological processes in CKM.

In a study conducted by Zheng et al. utilizing a high-fat diet mouse model, it was demonstrated that butyrate initially binds to GPR41/43 receptors in hepatocytes. This binding event subsequently initiates a CaMKII-mediated phosphorylation of HDAC1, leading to the activation of CREB [97]. This signaling cascade results in the suppression of lipogenic gene expression and the enhancement of fatty acid oxidation, exemplifying a scenario in which GPCR activation and HDAC inhibition operate within a singular linear pathway [97]. Similarly, the anti-inflammatory effects of butyrate have been ascribed to the activation of GPR109A and the inhibition of HDAC [120]. Distinguishing between these two mechanisms can be challenging. Functionally, GPCR signaling provides rapid cellular responses (minutes to hours), whereas HDAC inhibition induces longer-lasting transcriptional changes (hours to days). This temporal complementarity may allow butyrate to both quickly counteract acute inflammatory insults and maintain a sustained protective state.

The connection with the gut microbiota is not a separate axis but rather a positive feedback loop. Butyrate improves intestinal barrier integrity, at least partly, via GPR43-mediated upregulation of tight junction proteins (e.g., Zonula occludens-1 (ZO-1), occludin) [121]. A tighter barrier reduces systemic endotoxin translocation, thereby lowering the low-grade inflammation that fuels IR and endothelial dysfunction in CKM. Additionally, butyrate lowers colonic pH and consumes oxygen, creating an anaerobic niche that favors the growth of BPBs, including *F. prausnitzii* and *Roseburia* spp. [113,116,122,123]. This sets up a self-amplifying cycle: more butyrate creates a more favorable gut environment, which in turn supports greater butyrate production. In a CKD mouse model, Li et al. demonstrated that oral administration of *F. prausnitzii* improved renal function and reduced serum levels of uremic toxins [121]. This beneficial effect was nullified by GPR43 inhibition, suggesting that the improvement was mediated by butyrate’s interaction with host receptors rather than by the mere presence of the bacteria. Furthermore, this discovery proposes a potential strategy to circumvent the limited oral bioavailability of free butyrate by employing BPB as live biotherapeutic agents.

Clinically, the merging of these pathways enables butyrate to simultaneously affect multiple essential components of CKM. It improves insulin sensitivity via both HDAC-dependent and GPR43-dependent mechanisms [97,124]. Additionally, it suppresses metabolic inflammation by promoting anti-inflammatory cytokine profiles (via GPCR) and reducing pro-inflammatory gene transcription (via HDAC). Butyrate also restores gut homeostasis by lowering luminal pH, reducing oxygen, tightening the epithelial barrier, and suppressing iNOS-driven pathobiont expansion [113,116,122,123]. Furthermore, it has been shown to lower blood pressure, at least in animal models, by modulating RAAS activity and upregulating renal SCFA receptors [125]. Thus, the integrated network of butyrate action makes butyrate particularly suited for a complex, multi-organ syndrome like CKM, where single-target medications have often not worked.

### 3.2. An Integrative Perspective: Butyrate as a Multi-System Regulatory Hub in CKM

The substantial burden of CKM syndrome, coupled with compelling preclinical data, has spurred clinical and translational research into butyrate’s therapeutic role. This section moves beyond a simple cataloging of studies to critically appraise the human evidence linking butyrate and BPB deficiency to CKM components and evaluate the efficacy of butyrate-targeted interventions. The evidence is synthesized to illustrate a translational continuum: from observational associations (Summarized in Table 1 and Table 2) to interventional outcomes (Summarized in Table 3 and Table 4).

#### 3.2.1. Evidence for Butyrate and BPB Deficiency in CKM Syndromes: From Association to Potential Causality

##### Cardiovascular Diseases

CVDs, encompassing disorders of the heart and blood vessels such as coronary artery disease, heart failure, stroke, atherosclerosis, and hypertension, remain the leading cause of global mortality [163]. Patients with atherosclerotic cardiovascular disease, hypertension, heart failure, and atrial fibrillation show a marked reduction in the abundance of BPB in the gut, such as *Faecalibacterium*, *Roseburia*, and *Eubacterium* at the genus level, *R. intestinalis* and *F. prausnitzii* at the species level [126,133,164], and butyrate levels in serum and fecal. Additionally, a 12-month follow-up trial indicates that baseline fecal butyrate was inversely associated with prevalent hypertension [126]. A 10% elevation in fecal and serum butyrate levels from baseline was correlated with a reduction in systolic blood pressure, while a 10% increase in serum butyrate alone was correlated with a reduction in diastolic blood pressure.

##### Kidney Disease

CKD affects approximately 10–15% of the world’s population [13]. Its progression is frequently accompanied by hypertension, renal dysfunction, renal fibrosis, and ultimately progresses to ESRD [13,121]. Accumulating evidence underscores the role of gut microbiota (such as *Roseburia* spp. and *F. prausnitzii*) and their metabolites in the progression of CKD [10]. The most severe depletion is found in ESRD, where butyrate levels inversely correlate with renal function and markers of microinflammation [10,127,128]. This suggests a potential role of butyrate deficit in fueling the inflammatory milieu of CKD.

##### Hepatic Disease

Metabolic dysfunction-associated steatotic liver disease (MASLD), a leading cause of chronic liver disease worldwide affecting nearly 30% of adults, is closely interrelated with obesity, hyperlipidemia, diabetes mellitus, and CVD [151]. In MASLD, decreased fecal butyrate concentrations correlate with histological disease severity, and gut dysbiosis is marked by a loss of BPB families like *Coprococcus*, *Faecalibacterium*, *Ruminococcaceae*, and *Lachnospiraceae* [129,165,166].

##### Metabolic Diseases

Metabolic diseases mainly include obesity, diabetes, hyperuricemia, metabolic syndrome, and hyperlipidemia. In patients with obesity, a significant reduction in the relative abundance of key BPB, including *F. prausnitzii*, *A. hadrus*, *R. intestinalis*, *Ruminococcus gnavus*, *B. luti*, *B. wexlerae*, *E. hallii*, and *E. ventriosum*, was observed [12,130,142]. Clinical studies also showed that butyrate was negatively associated with body mass index, visceral fat area, and blood pressure in patients with obesity [131,132]. Patients with T2D exhibit a marked reduction in BPB, such as *F. plautii*, *Anaerostipes caccae*, *C. paraputrificum*, and *C. butyricum*, linking specific gut microbiota compositional changes to the disease [12,143,167]. Consistently, butyrate was negatively associated with postprandial blood glucose in patients with T2D [131]. Gut microbiota dysbiosis in hyperuricemia and hyperlipidemia is characterized by reductions in *Faecalibacterium*, *Coprococcus*, and *Enterococcus*, and an expansion of opportunistic pathogens in patients [147,168].

Nevertheless, while these observational studies robustly establish an association, they cannot confirm causality. The reductions in BPB and butyrate could be a consequence of disease-related dietary changes, systemic inflammation, or medication use. However, the consistency of the signal across geographically distinct cohorts and its correlation with disease severity strengthen the hypothesis of a pathophysiological contribution.

#### 3.2.2. Interventional Evidence: Translating Mechanisms into Outcomes

**Figure 3:**
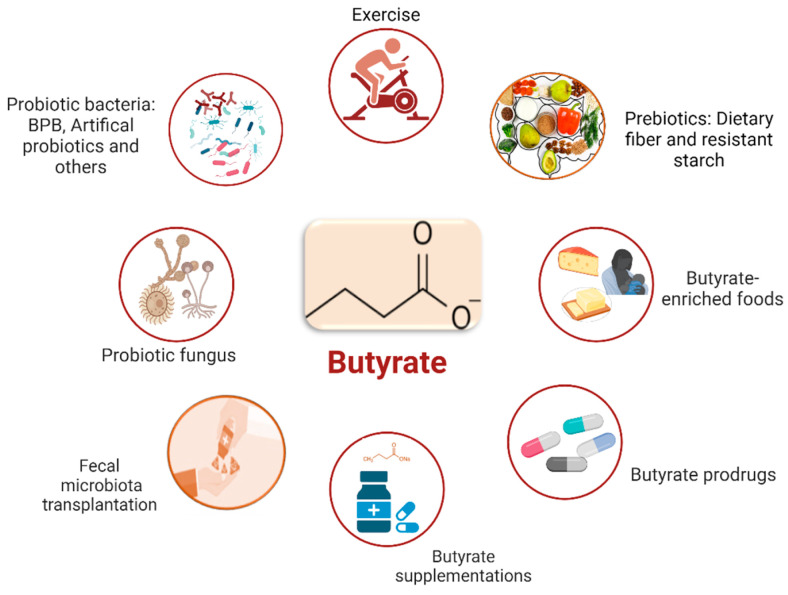
Approaches to butyrate production.

Intervention studies provide stronger evidence for the therapeutic potential of butyrate and BPB. These approaches fall into two categories: direct supplementation, indirect modulation of the gut microbiome, and prodrugs (Figure 3).

##### Direct Butyrate Supplementation

Human trials of direct butyrate administration, primarily with sodium butyrate, show promising yet nuanced results.

Randomized controlled trials demonstrate that the butyrate supplementation increased the level of serum butyrate and significantly decreased the systolic blood pressure [148,149]. Animal studies indicated that the underlying mechanisms are modulation of the renin-angiotensin system, inhibition of vascular inflammation, improvement of endothelial function, and alterations in microbiome composition [88,125,169].

In patients with MASLD, the supplementation of butyrate significantly reduced serum TMAO, total cholesterol, triglyceride, and fatty liver index [150,151]. The potential mechanism is that butyrate supplementation inhibited HDAC2, thereby upregulating hepatic glucagon-like peptide-1 (GLP-1) receptor expression, improving systemic energy metabolism, and reducing hepatic lipid accumulation in MASLD models [170]. As a gut-derived incretin hormone, GLP-1 enhances insulin sensitivity and exerts anti-inflammatory and hypoglycemic effects, which are protective in MASLD [171,172].

In patients with T2D, randomized, double-blind, placebo-controlled trials show that oral butyrate (600 mg/day) for 45 days reduced hip circumference and diastolic blood pressure, elevated postprandial GLP-1 levels, and enhanced antioxidant capacity [154,173]. Two additional long-term trials indicated that butyrate supplementation improved HbA1c and postprandial blood glucose levels in T2D patients [155,156]. In patients with obesity, randomized trials in both adults and children report a significant reduction in body mass index and waist circumference [152,153,157]. In patients with metabolic syndrome, oral butyrate supplementation (4 g/day) for 4 weeks has been shown to decrease HbA1c, total cholesterol, and triglycerides [157].

However, clinical evidence for direct butyrate benefits in CKD and hyperuricemia remains primarily preclinical. A major translational challenge is the pharmacokinetic profile of butyrate, including its rapid absorption and metabolism in the upper GI tract, and its unpleasant odor, which limits its oral bioavailability and tolerability at high doses.

##### Indirect Enhancement via Gut Microbiota

Given that the majority of endogenous butyrate in humans is derived from microbial fermentation of non-digestible carbohydrates like dietary fiber and resistant starch [174]. A primary strategy for boosting butyrate is to modulate the gut ecosystem. This involves increasing the abundance of BPB and/or providing them with necessary substrates.

*Probiotics and engineered strains*

The administration of specific probiotic strains has shown promise. Supplementation with the BPB *A. soehngenii* in prediabetic adults significantly lowered diastolic blood pressure, improved glycemic control, and other markers of cardio-metabolic health [160]. Non-butyrate-producing bacteria *Bifidobacterium bifidum* CCFM16 could increase fecal butyrate levels and enrich butyrate-associated *Clostridia* in individuals with chronic constipation [175]. Similarly, a 5-strain probiotic formulation significantly increased plasma butyrate and improved glycemic control (reduced HbA1c) in patients with T2D over 12 weeks [176]. A more advanced frontier involves the use of engineered probiotics, designed to directly or indirectly enhance butyrate production. A notable example is a genetically modified *Bacillus subtilis* SCK6, which was shown to boost butyrate yield by 3.8-fold compared to its native counterpart [177]. Additionally, *Eurotium cristatum*, a potential probiotic fungus from Fuzhuan brick tea, alleviated obesity by modulating BPB and increasing butyrate level [178].

*Fecal microbiota transplantation (FMT)*

FMT has emerged as a powerful tool for rapidly altering the gut microbial community. In patients with obesity and type 2 diabetes, FMT led to decreased LDL-C and a significant, sustained increase in key BPB, including *F. prausnitzii*, *R. hominis*, and various *Eubacterium* and *Coprococcus* species [179].

*Prebiotics*

Prebiotics, defined as selectively fermented ingredients that confer health benefits, directly serve as substrates for BPB. Supplementation with fructo-oligosaccharides, oats, omega-3 fatty acids, nonstarch polysaccharides, and inulin has consistently been shown to increase fecal butyrate levels [173,180,181,182,183]. These interventions have yielded positive clinical endpoints, including inhibition of pyroptosis in T2D. Furthermore, overarching dietary patterns profoundly influence butyrogenesis.

*Administration of lifestyle*

Lifestyle interventions, such as exercise and dietary modulation, also exert beneficial effects on butyrate production. An 8-week intervention comparing a Mediterranean diet to a Western-type diet in obese subjects revealed that the Mediterranean diet group exhibited a significantly higher postprandial plasma butyrate response and an increased abundance of BPB, like *I. butyriciproducens* and *R. hominis* [184]. Independent of diet, exercise training in murine models has also been demonstrated to increase fecal butyrate levels and enrich BPB, such as *R. hominis*, *F. pausnitzii*, and *Ruminococcaceae* [185].

##### Indirect Enhancement via Prodrugs and Pharmacological Agents

Butyrate prodrugs represent an innovative pharmacological strategy to address the rapid metabolism of pure butyrate and its unpleasant odor. Tributyrin, a stable glycerol ester, boosts plasma butyrate in rodents and may outperform native butyrate in suppressing colorectal cancer cells [186,187]. Arginine butyrate induced antitumor responses in 10/15 patients with refractory Epstein–Barr virus-associated lymphoid malignancies [188]. Notably, existing drugs like the antidiabetic acarbose significantly increase fecal butyrate [189].

Despite this promise, the intervention landscape is heterogeneous. Study durations are often short-term, doses vary, and outcome measures are diverse, making meta-analyses difficult. The most compelling human data exist for metabolic endpoints (glycemia, obesity), whereas evidence for hard cardiovascular or renal outcomes (e.g., myocardial infarction, CKD progression) remains limited. Future trials require longer follow-up, standardized butyrate assessment, and clinically meaningful endpoints.

## 4. Butyrate-Producing Bacteria in CKM Syndrome

**Figure 4:**
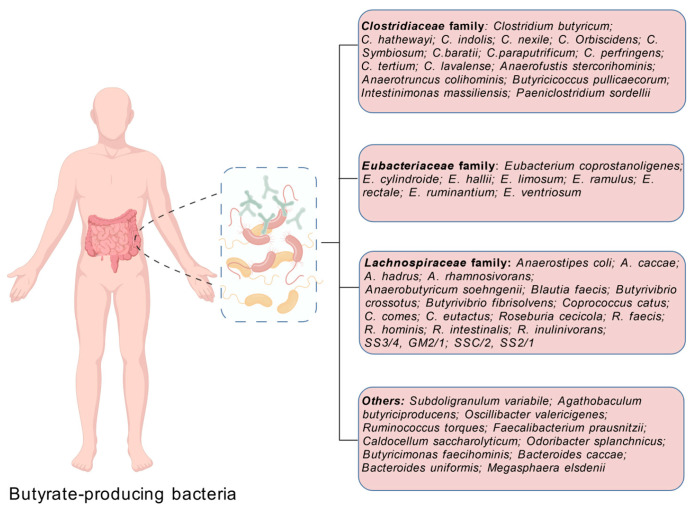
BPB found in the human gastrointestinal tract.

BPB are a type of bacteria with butyrate as its primary metabolite, distributed in the human gut and oral tract, and can also be found in animals’ gut, plants, soil, and agricultural lagoons. Most BPB are Gram-positive and strictly anaerobic [190]. BPB mainly belongs to four families: *Clostridiaceae*, *Eubacteriaceae*, *Lachnospiraceae*, and *Ruminococcaceae*. Of note, some members of *Oscillospiraceae* and *the Bacteroidaceae* family can also secrete butyrate, such as *Oscillibacter valericigenes* NBRC 101213T and *Bacteroides uniformis* [190]. Over fifty BPBs were found in the human gut and potentially play a positive role in human health (Figure 4).

**Figure 5:**
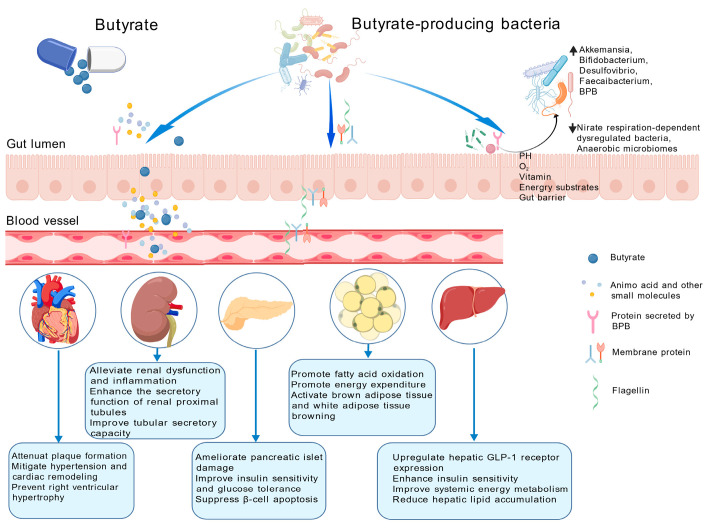
Effect of butyrate and BPB in the CKM syndrome. Butyrate plays a protective role in CKM syndrome-associated diseases by modulating metabolic organs. Besides butyrate, BPB has a positive effect on the CKM syndrome through bacterial components, the production of other functional metabolites, or the regulation of gut microbiota composition.

For nearly twenty years, numerous studies have focused on BPBs as a novel type of probiotic. As shown in Table 2 and Table 4, BPB depletion in the host gut is associated with CKM progression, and BPB supplementation could improve CKM syndrome. Except for butyrate, BPB can also have a positive effect on the CKM syndrome through butyrate-independent pathways, including the production of butyrate-independent functional metabolites, bacterial components, and the regulation of gut microbiota composition (Figure 5).

Microbial anti-inflammatory molecule (MAM), a protein produced by *F. prausnitzii*, possesses beneficial effects in diabetes [191]. MAM can restore the structure and function of the intestinal barrier and improve gut inflammation via regulating the tight junction pathway and upregulating the expression of ZO-1, which is an important structural protein of tight junctions [191,192]. Acetate and propionate, major products of *A. muciniphila*, which also belong to the SCFA family [193,194]. Acetate derived from microbiota mainly affects cognitive function, and the long-term deficiency of acetate could induce cognitive impairment in Type 1 diabetes [195]. Similarly, microbial-derived propionate improved heart function and prevented cardiac fibrosis through activating GPR41/GPR43 [196]. Besides SCFA and MAM, BPB can also form lactate, formate, hydrogen gas, and carbon dioxide, which may be involved in human health [117].

Another direct way BPB exerts influence is through its cell components. *C. butyricum* is a producer of butyrate and can be isolated in human and animal intestines and found in soil [197]. Studies indicated that supplementation with *C. butyricum* protected against IR and metabolic inflammation and reduced lipogenesis through the bacterial wall components in obesity models [197,198]. However, the mechanism by which bacterial wall components exert a beneficial effect remains unclear. In addition, investigators also observed that the flagellin of *R. intestinalis*, another member of BPB from the *Roseburia genus*, inhibited inflammation-induced apoptosis in a mouse model [199].

Similarly, BPB intervention could regulate gut microbiota composition by enriching beneficial flora and inhibiting harmful flora. After the intervention of *C. butyricum* in diet-induced diabetic mice, the abundance of *Clostridiaceae*, *Bacteroidaceae*, *Porphyromonadaceae*, *Rikenellaceae*, *Deferribacteraceae*, *Lactobacillaceae*, and *Helicobacteraceae* families increased significantly, while the *Erysipelotrichaceae* family decreased [200]. There are several possible mechanisms for this phenomenon. The first potential reason is the syntrophic relationship between BPB and other probiotics. Although there is nutrient competition among gut bacterial communities, the metabolites of many bacteria may serve as essential energy sources for other bacteria, and their growth depends on the secretions of other bacteria [201]. For instance, keystone mucolytic bacteria, such as *A. muciniphila*, can decompose mucin glycans to produce oligosaccharides (mainly galactose, fucose, and mannose) and SCFAs (1,2-propanediol, acetate) when it is cultured with butyrate producer *E. hallii* [202]. These oligosaccharides and SCFA could promote the growth of *E. hallii* as energy substances and substrates. In turn, the Vitamin B12 analogue produced by *E. hallii* also promotes the growth and metabolism of *A. muciniphila* [202]. Not only that, the metabolites of BPB, such as butyrate, lactate, formate, and carbon dioxide, will regulate the pH of the intestines and create an acidic environment to boost the growth of bacteria that prefer mild acid conditions, such as *R. intestinalis*, *E. rectale*, and *F. prausnitzii* [114,115,203], and inhibit the growth of pathogenic bacteria, such as *Clostridioides difficile* [204].

## 5. Concluding Remarks and Future Perspectives

In this review, we have integrated current evidence to propose that butyrate and BPB play a protective role in CKM syndrome. Our synthesis leads to three principal observations. First, butyrate acts through multiple interconnected mechanisms, including HDAC inhibition, GPCR activation, and gut ecosystem remodeling, that collectively target the core pathophysiological features of CKM, including IR, metabolic inflammation, oxidative stress, endothelial dysfunction, RAAS overactivation, and gut dysbiosis. Second, depletion of BPB, particularly *F. prausnitzii* and *Roseburia* spp., is a consistent ecological signature across CKM conditions and correlates with disease severity, suggesting that loss of these bacteria may be an independent driver of disease progression rather than a mere consequence. Third, while direct butyrate supplementation has shown promising effects on surrogate endpoints such as glycemic control and blood pressure, its clinical utility is limited by poor bioavailability, short half-life, and lack of long-term safety data. Strategies that enhance endogenous butyrate production through targeted probiotics, prebiotics, dietary patterns, and exercise may be more feasible for sustained intervention.

Several limitations of the current literature warrant acknowledgment. Most human studies are small, short-term, and heterogeneous in design, with few examining hard clinical endpoints such as major adverse cardiovascular events, renal function decline, or mortality. The optimal dosing, duration, and route of butyrate administration remain undefined, and safety data beyond six months are virtually absent. Moreover, whether butyrate deficiency is a causal factor or an epiphenomenon in CKM pathogenesis has yet to be established through longitudinal or Mendelian randomization studies.

Looking forward, we propose the following priorities for translational research: (1) large-scale, long-term randomized controlled trials that stratify patients by CKM stage and baseline BPB abundance; (2) inclusion of multi-organ endpoints (e.g., eGFR, albuminuria, cardiac imaging, glucose tolerance) to capture the syndrome’s integrated nature; (3) development of validated biomarkers for butyrate status to guide patient selection and monitor response; (4) systematic evaluation of combination regimens that synergistically boost endogenous butyrate; and (5) human tissue-based mechanistic studies to confirm the relevance of HDAC and GPCR pathways identified in animal models.

In conclusion, preclinical data collectively support that butyrate and BPB serve as a theoretically innovative and mechanistically reasonable therapeutic target for CKM syndrome, yet robust large-scale human intervention trials remain scarce. Interpreting CKM syndrome within the gut-butyrate-CKM axis framework facilitates a shift from isolated single-disease treatment to holistic microbiota-targeted intervention. Nevertheless, translating these laboratory findings into routine clinical practice still requires large, rigorously designed long-term human trials to validate efficacy and safety. At present, high-fiber dietary intervention is a safe, evidence-based measure whose partial protective effects are likely mediated by elevated endogenous butyrate levels. Direct oral supplementation with butyrate or BPB cannot yet be recommended for general clinical application and should be restricted to standardized clinical research only.

## Figures and Tables

**Table 1:** Summary of clinical studies investigating the association between butyrate and CKM syndrome.

Disease	Sample Size	Study Design	Key Results	Reference
Hypertension	121	12-month follow-up in overweight/obese cancer survivors	Baseline fecal butyrate is inversely associated with prevalent hypertension. A 10% increase in fecal butyrate was associated with decreased systolic BP; 10% increase in serum butyrate associated with decreased SBP and DBP.	[126]
CKD	54	SCFA levels measured in 54 CKD patients at different stages	Fecal butyrate reduced as CKD progressed, closely correlating with serum creatinine, blood urea nitrogen, and eGFR.	[127]
CKD	190	127 CKD patients and 63 healthy controls from China	Butyrate was nearly three times higher in healthy controls than in CKD5 subjects. Serum SCFA levels are significantly higher in controls; butyrate level in CKD5 were less than half of those in the controls.	[128]
NAFLD	104	21 healthy controls and 83 NAFLD patients	Butyrate levels were significantly lower in NAFLD patients than in non-NAFLD individuals.	[129]
Type 1 diabetes + overweight/obesity	45	SCFA and SCFA producers were detected	SCFA producers inversely associated with % body fat or BMI, and positively associated with % lean mass.	[130]
T2D with obesity	20	8 healthy controls; 12 T2D with obesity	Butyrate concentration significantly increased after substantial weight loss; negatively associated with BMI, visceral fat area, PBF, and 2 h PG.	[131]
Obese pregnant women	205	205 overweight/obese pregnant women; fecal and fasting serum samples at 16 weeks gestation	Abundance of butyrate-producing bacteria and butyrate production significantly negatively associated with blood pressure and plasminogen activator inhibitor-1 levels.	[132]

**Table 2:** Summary of clinical studies investigating the association between BPB and CKM syndrome.

Disease	Sample Size	Study Design	Results	Reference
Hypertension	196	41 healthy controls; 56 pre-hypertension; 99 primary hypertension	↓ *Faecalibacterium*, *Roseburia*	[133]
ACVD	405	187 healthy controls; 218 ACVD	↓ *R. intestinalis*, *F. prausnitzii*	[134]
CAD	201	40 healthy controls; 161 CAD	↓ *Faecalibacterium*, *Roseburia*	[135]
AD-T2D	32	16 CAD-NT2D; 16 CAD-T2D	↓ *F. prausnitzii*	[11]
Hypertension	61	32 controls; 29 non-treated hypertension	↓ *F. prausnitzii*, *R. hominis*	[136]
Heart Failure	827	510 controls; 317 Heart failure	↓ *E. rectale*, *F. prausnitzii*	[137]
Atrial fibrillation	100	50 controls; 50 atrial fibrillation	↓ *Faecalibacterium*, *Oscillibacter*	[138]
CKD	85	20 healthy controls; 65 CKD	↓ *Roseburia*, *F. prausnitzii*	[10]
CKD	320	210 healthy controls; 110 CKD	*↓ Roseburia*, *Faecalibacterium*, *Blautia*	[139]
MASLD	189	87 Healthy controls; 102 MASLD	↓ *Faecalibacterium*, *Subdoligranulum*	[9]
T2D-MASLD	255	73 T2D; 182 T2D-MASLD	↓ *Butyricicoccus*, *Ruminococcus*, *Butyricimonas*	[140]
Obesity-MASLD	181	78 obesity; 103 obesity-MASLD	↓ *F. prausnitzii*	[141]
Obesity	51	16 healthy controls; 35 Obesity	↓ *Blautia luti*, *Blautia wexlerae*, *Eubacterium hallii*	[142]
Obesity + T2D	229	101 lean controls; 128 obese subjects	*↓ F. prausnitzii*, *Anaerostipes hadrus*, *R. intestinalis*, *R. hominis*, *Eubacterium eligens*	[12]
T2D	291	193 healthy controls; 98 T2D	Genus-level: ↓ *Collinsella*, *Anaerostipes*, *Clostridium*; Species-level: ↓ *Cellulosilyticum ruminicola*, *Clostridium paraputrificum*, *Clostridium butyricum*, *Ruminococcus lactaris*	[143]
T2D	80	40 healthy controls; 40 T2D	↓ *F. prausnitzii*	[144]
Hyperuricemia	356	178 controls; 178 hyperuricemia	Genes-level: ↓ *Lactobacillus*, *Bacteroides*, *Enterococcus*; Species-level: ↓ *Clostridium leptum*, *F. prausnitzii*, *C. butyricum*	[145]
Hyperuricemia	107	57 controls; 50 hyperuricemia	↓ *Ruminococcus*	[146]
Hypercholesterolemic	57	30 controls; 27 hypercholesterolemic	↓ *Faecalibacterium*	[147]

↓: Decrease of the abundance of butyrate-producing bacteria at the genus or species level.

**Table 3:** Summary of clinical studies assessing the effect of butyrate supplementation in CKM syndrome.

Disease	Study Design	Intervention	Primary Outcome	Main Limitations	Reference
Hypertension	Proof-of-concept randomized controlled trial	Intervention group (*n* = 10): Randomly self-administered a low dose (5 mmol/L) and a high dose (80 mmol/L) butyrate enema 7 days apart; Control group (*n* = 10): Placebo dose of 5 mmol/L butyrate was used in 10 controls with normal BP to keep participants and researchers blinded to treatment	Daytime SBP was significantly lower after the 80 mmol/L enema compared with the 5 mmol/L enema (132.9 ± 12.64 vs. 137.5 ± 13.46, *p* = 0.034).	Not reported	[148]
Hypertension	Randomized, placebo-controlled, double-blind cross-over trial	20 hypertensive participants were randomized to 40 g/day HAMSAB or placebo, with 3 weeks per arm and a 3-week washout period in between	HAMSAB treatment induced a clinically relevant 24 h SBP reduction (−4.8 mmHg, *p* = 0.029), increased acetate and butyrate levels (14-fold, *p* = 0.0178), and expanded the prevalence of SCFA producers	Small sample size; Lack of long-term follow-up; The differences in dietary fiber content between treated and control groups.	[149]
MASLD	Randomized double-blind placebo-controlled clinical trial	Butyrate group (*n* = 25): Tablets (calcium butyrate (500 mg/tablet), zinc gluconate (zinc 5 mg/tablet), and vitamin D3 (500 IU/tablet)), for 12 weeks; Control group (*n* = 25): Placebo, for 12 weeks	Compared to the placebo group, the active treatment group exhibited significant improvements in the FLI (69.5 ± 14.7 vs. 73.3 ± 15.6), TC (221 ± 11 vs. 228 ± 12), and TG (207 ± 23 vs. 218 ± 23), all *p* < 0.05.	Non-invasive NAFLD diagnosis; Relatively small sample size; Short intervention duration; Unassessed dietary–microbiota interactions	[150]
MASLD	Single-center, randomized clinical trial	Butyrate group (*n* = 121): 1000 mg/day NaB, for 12 weeks; Control group (*n* = 60): 1000 mg/day calcium butyrate, for 12 weeks	NaB markedly reduced serum TMAO (*p* = 0.021) and FLI (*p* = 0.047), whereas calcium butyrate lowered fecal calprotectin (*p* = 0.031).	Short treatment duration; Limited sensitivity of controlled attenuation parameter; Absence of longitudinal microbiome profiling	[151]
Pediatric obesity	A randomized, quadruple-blind, placebo-controlled trial	Butyrate group (*n* = 27): Oral sodium butyrate at 20 mg/kg body weight per day, for 6 months; Control group (*n* = 27): Placebo, for 6 months	Butyrate-treated children had a higher rate of BMI decrease (96% vs. 56%, absolute benefit increase, 40%; 95% CI, 21% to 61%; *p* < 0.01), along with decreased changes in WC (−5.07 cm (95% CI, −7.68 to −2.46 cm; *p* < 0.001)), insulin levels (−5.41 μU/mL (95% CI, −10.49 to −0.34 μU/mL; *p* = 0.03)), HOMA-IR (−1.14 (95% CI, −2.13 to −0.15; *p* = 0.02)), ghrelin levels (−47.89 μg/mL (95% CI, −91.80 to −3.98 μg/mL; *p* < 0.001))	Absence of key objective measures; No objective quantification of physical activity	[152]
Obesity	Triple-blind placebo-controlled randomized clinical trial	Butyrate group (*n* = 25): 600 mg/day NaB + hypo-caloric diet, for 8 weeks; Control group (*n* = 25): placebo capsules + hypo-caloric diet, for 8 weeks	NaB increased PGC-1α and UCP-1 gene expression (PGC-1α fold change: 1.84 ± 0.44 vs. 0.8 ± 0.2, *p* = 0.049; UCP-1 fold change: 1.82 ± 0.42 vs. 0.48 ± 0.19, *p* = 0.012) and decreased weight (2.82 (1.66, 3.98), *p* < 0.001), BMI (0.96 (0.49, 1.42), *p* < 0.001), and waist circumference (−4 (−8.75, −2), *p* < 0.001), fasting blood sugar (−3 (−9.5, 0.5), *p* = 0.017), TC (−13 (−23.5, −7.5), *p* = <0.001), TG (−38 (−55, −7.5), *p* < 0.001), LDL-C (−15 (−29.6, 1.1), *p* < 0.001), and increased HDL-C (10 (0.5, 18), *p* < 0.001)	Inability to assess NaB effects in human visceral tissues; Possibly insufficient intervention duration	[153]
Overweight participants with T2D	Randomized controlled trial	NaB group (*n* = 15): 600 mg/day NaB, for 45 days; Inulin group (*n* = 14): 10 g/day Inulin, for 45 days; Inulin with NaB group (*n* = 15): 600 mg/day NaB + 10 g/day Inulin, for 45 days; Placebo group (*n* = 15): Placebo, for 45 days	NaB intervention significantly reduced diastolic blood pressure (78.33 ± 8.38 vs. 85.67 ± 8.42, *p* = 0.013); Treatment with NaB + inulin significantly reduced fasting blood sugar (158.86 ± 42.39 vs. 176.86 ± 56.10, *p* = 0.049), waist-to-hip ratio (0.88 ± 0.05 vs. 0.89 ± 0.05, *p* = 0.020), and WC (93.84 ± 8.77 vs. 95.58 ± 9.37, *p* = 0.011)	Short intervention duration; Small sample size; Lack of serum SCFAs measurement; Lack of serum endotoxin concentration assessment; Lack of inflammatory cytokine profiling in serum.	[154]
T2D	Randomized triple-blind, placebo-controlled trial	Butyrate group (*n* = 21): 600 mg/day NaB, for 6 weeks; Placebo group (*n* = 21): placebo, for 6 weeks	NaB administration significantly reduced SBP (128.25 ± 9.07 vs. 136.25 ± 18.12, *p* = 0.016), DBP (77.75 ± 7.69 vs. 85.25 ± 7.69, *p* = 0.002), and 2 h PG (165.00 (96.00–318.00) vs. 220.81 (103.00–347.00), *p* = 0.016)	SCFA measurement omitted; No dose–response evaluation; Relatively short study duration	[155]
T2D	Prospective, randomized, placebo-controlled double-blind study	Butyrate group (*n* = 29): 1.5 g/day butyrate, for 12 weeks; Placebo group (*n* = 23): Placebo, for 12 weeks	Butyrate-treated patients showed a slight but significant improvement in BMI (27.92 ± 4.0 vs. 28.54 ± 4.27) and HbA1C levels (6.00 ± 1.1 vs. 6.38 ± 1.24)	Not reported	[156]
Metabolic syndrome	Double-blind randomized controlled intervention trial	Butyrate group (*n* = 12): a single autologous fecal transplantation, serving as placebo, followed by 4 g of oral NaB tablets once daily for 4 weeks; Post-RYGB group (*n* = 12): a single post-RYGB donor fecal transplantation followed by similar daily amounts of placebo tablets for 4 weeks	The decrease in HbA1c (37 (34–44) vs. 40 (35–45), *p* = 0.04), total cholesterol (5.1 ± 0.8 vs. 5.5 ± 0.8, *p* = 0.04), and TG (1.2 (0.9–1.4) vs. 1.4 (1.1–1.7), *p* = 0.03) was observed in the butyrate group	Small sample size and ethnically homogeneous cohort; Single-donor FMT may not suffice for durable effects; Ethical restrictions precluded long-term imaging assessments; Food reward-related outcomes not examined	[157]

**Table 4:** Summary of clinical studies assessing the effect of BPB supplementation in CKM syndrome.

Probiotics	Disease	Study Design	Intervention	Primary Outcome	Main Limitations	Reference
*Intestinimonas butyriciproducens*	Overweight or obese	Double-blind, randomized, placebo-controlled (phase 1)/Open-label pilot study/(phase 2)/	Phase 1: *I. butyriciproducens* (10^5^ CFU/day) or placebo, for 12 weeks Phase 2: Subjects in placebo group start the treatment (10^5^ CFU/day), and the other group increases the dose (10^8^ CFU/day), for 14 weeks	Treated patients during phase 1 had a significant improvement in glucose-insulin metabolism (*p* < 0.05); Lipid profile ameliorated in patients treated at a low dose and then at a high dose, particularly decreasing TG (All *p* < 0.05)	Proof-of-concept study with limited sample size and short observation period	[158]
*C. butyricum* CGMCC 0313.1	MS in patients with schizophrenia	Open-label pilot study	Intervention group (*n* = 52): *C. butyricum* CGMCC 0313.1 (2 capsules per dose, 3 times daily), for 12 weeks Control group (*n* = 48): lifestyle interventions, for 12 weeks	The intervention group showed more pronounced reductions in obesity indices, BS levels, lipid profiles, and BP	Small sample size and short intervention duration; Uncontrolled antipsychotic regimens; Lack of mechanistic exploration	[159]
*Anaerobutyricum soehngenii*	Prediabetic insulin-resistant	Double-blind, randomized placebo-controlled trial	Intervention group (*n* = 49): *A. soehngenii* strain CH-106 (10^9^ Active Fluorescent Units, daily), for 12 weeks Control group (*n* = 49): Placebo, for 12 weeks	The intervention group showed significantly reduced glycemic variability and improved glycemic control, including reduced serum HbA1c levels; DBP was significantly reduced in all *A. soehngenii*-treated subjects (approximately 1 mmHg, *p* < 0.05)	Cross-site baseline heterogeneity; Modest sample size; Short intervention duration	[160]
*A. soehngenii*	T2D	Randomized, double-blind, placebo-controlled trial	Intervention group (*n* = 12): once-daily oral treatment with *A. soehngenii* L2–7, for 14 days Control group (*n* = 12): Placebo arm, for 14 days	*A. soehngenii* significantly improved glycemic variability (Standard deviation was reduced by 7.96% (FDR corrected *p* = 0.034), MAGE was reduced by 16.82% (FDR corrected *p* = 0.027), and MODD was reduced by 3.9% (FDR corrected *p* = 0.034)) and MAP (MAP was reduced by 10.24% (*p* = 0.04))	Short intervention period (proof-of-concept, limited duration); Small sample size; Concomitant metformin therapy (potential confounding effect on gut microbiota, though authors argue it enhances generalizability)	[161]
*C. butyricum*	NAFLD	Randomized, open-label pilot study	Intervention group (*n* = 48): *C. butyricum* capsules (400 mg/time) combined with rosuvastatin (twice a day, 10 mg/time), for 6 months Control group (*n* = 48): rosuvastatin (twice a day, 10 mg/time), for 6 months	The intervention group had remarkably lower levels of TC (5.21 ± 1.18 vs. 4.05 ± 1.03), TG (4.08 ± 1.03 vs. 3.02 ± 0.89), free fatty acids (0.87 ± 0.37 vs. 0.35 ± 0.12), total bilirubin (37.24 ± 5.69 vs. 30.20 ± 5.38), direct bilirubin (18.91 ± 5.23 vs. 11.24 ± 4.53), alanine aminotransferase (36.88 ± 7.93 vs. 26.37 ± 6.55), aspartate aminotransferase (42.55 ± 9.22 vs. 31.58 ± 9.64), procollagen III peptide (183.46 ± 13.27 vs. 108.83 ± 13.14), collagen-IV (98.14 ± 9.38 vs. 74.45 ± 7.07), hyaluronic acid (232.46 ± 23.79 vs. 105.53 ± 12.37), and laminin (153.53 ± 12.65 vs. 87.42 ± 9.38), and lower levels of TNF-α (25.63 ± 3.56 vs. 17.37 ± 3.54), CRP (3.26 ± 0.68 vs. 1.24 ± 1.36), and IL-6 (12.53 ± 2.38 vs. 6.82 ± 1.75) in serum, All *p* < 0.001	Not reported	[162]

## Data Availability

No new data were created or analyzed in this study. Data sharing is not applicable to this article.

## Abbreviations

The following abbreviations are used in this manuscript:

2 h PG	2-hour postprandial blood glucose
*A. hadrus*	*Anaerostipes hadrus*
*A. Muciniphila*	*Akkermansia. muciniphila*
*A. soehngenii*	*Anaerobutyricum soehngenii*
ACVD	Atherosclerotic cardiovascular disease
AHR	Aryl hydrocarbon receptor
*B. luti*	*Blautia luti*
*B. wexlerae*	*Blautia wexlerae*
BMI	Body mass index
BP	Blood pressure
BPB	Butyrate-producing bacteria
BS	Blood sugar
*C. butyricum*	*Clostridium butyricum*
*C. paraputrificum*	*Clostridium paraputrificum*
CAD	Coronary artery disease
CD14	Cluster of differentiation 14
CFU	Colony-forming unit
CKD	Chronic kidney disease
CKM	Cardiovascular–kidney–metabolic
CRP	C-reactive protein
CVD	Cardiovascular disease
CYP1B1	Cytochrome P450 family 1 subfamily B member 1
DBP	Diastolic blood pressure
DKD	Diabetic kidney disease
*E. hallii*	*Eubacterium hallii*
*E. rectale*	*Eubacterium rectale*
eGFR	estimated glomerular filtration rate
ESRD	End-stage renal disease
*F. plautii*	*Flavonifractor plautii*
*F. prausnitzii*	*Faecalibacterium prausnitzii*
FLI	Fatty liver index
FMT	Fecal microbiota transplantation
GGT	γ-glutamyl transferase
GLP-1	Glucagon-like peptide-1
GPCR	G protein-coupled receptor
HbA1c	Glycated hemoglobin A1c
HDACs	Histone deacetylases
HDL-C	High-density lipoprotein cholesterol
HOMA-IR	Homeostasis model assessment of insulin resistance
hs-CRP	High-sensitivity C-reactive protein
*I. butyriciproducens*	*Intestinimonas butyriciproducens*
IL-6	Interleukin-6
IR	Insulin resistance
LPS	Lipopolysaccharide
MAGE	Mean amplitude of glycemic excursion
MAM	Microbial anti-inflammatory molecule
MAP	mean arterial blood pressure
MASLD	Metabolic syndrome-associated steatotic liver disease
MODD	Mean of daily differences
MyD88	Myeloid differentiation primary response 88
NaB	Sodium butyrate
NAFLD	Non-alcoholic Fatty Liver Disease
NF-κB	Nuclear factor kappa-B
NLRP3	NOD-like receptor thermal protein domain associated protein 3
PBF	Percent body fat
PGC-1α	Peroxisome proliferator-activated receptor γ coactivator 1-alpha
*R. hominis*	*Roseburia hominis*
*R. intestinalis*	*Roseburia intestinalis*
RAAS	Renin–angiotensin–aldosterone system
ROS	Reactive oxygen species
SBP	Systolic blood pressure
SCFAs	Short-chain fatty acids
T2D	Type 2 diabetes
TC	Total cholesterol
TG	Triglycerides
TLR4	Toll-like receptor 4
TMAO	Trimethylamine N-oxide
TNF-α	Tumor necrosis factor-alpha
WC	Waist circumference
ZO-1	Zonula occludens-1
